# Suspected autoimmune encephalitis in the presence of neuropsychiatric symptoms. The importance of differential diagnosis

**DOI:** 10.1192/j.eurpsy.2025.1257

**Published:** 2025-08-26

**Authors:** E. Talaya Navarro, L. Martín de Francisco Murga, L. Gallardo Borge, M. E. Gómez Fernández, S. N. Pecheykin Pajomov, M. Teijeiro Rebolo, A. Peman Alcazar

**Affiliations:** 1 Psiquiatría, Complejo Asistencial de Segovia, Segovia, Spain

## Abstract

**Introduction:**

Autoimmune encephalitis is a new and increasingly well-described entity. The most common is encephalitis caused by antibodies against the N-methyl-D-aspartate receptor on the surface of neurons (NMDAR encephalitis). It is a predominant entity in women and young patients and it is often associated with ovarian teratomas, neuroblastomas, Hodgkin’s lymphomas and others.

NMDAR encephalitis can manifest with a diverse range of neurological and psychiatric symptoms (personality change, anxiety, insomnia, confusion, attentional and short-term memory deficits, emotional lability, psychotic symptoms, language impairment, fluctuations in the level of consciousness, seizures and dysautonomia).

**Objectives:**

Clinical review of Anti-NMDAR Encephalitis for differential diagnosis with Functional Neurologic Disorder.

**Methods:**

Clinical case and literature review.

**Results:**

We present the clinical case of a 27-year-old woman with a history of depression and anancastic personality disorder. The patient went to the Emergency Department different days presenting both neurological (facial paresthesias, hypoesthesia, weakness, high-intensity occipital headache, dizziness, loss of consciousness and anterograde amnesia) and psychiatric symptoms (obsessive thoughts, anxiety, visual and auditory hallucinations). She was admitted to the Neurology Unit. Complementary tests were performed: EEG, cranial CT scan, MRI, lumbar puncture, blood tests (including tumour markers) and urinalysis, founding no alterations suggestive of encephalitis or other systemic pathologies. She was also evaluated by the Psychiatry service. The patient described that as a result of a recent change in her job she presented emotional lability, obsessive ruminative ideas and anxiety. Treatment with Sertraline 100mg/day and Lorazepam 1mg/8 hours was started.

After ruling out autoimmune encephalitis, the patient was diagnosed with Functional Neurologic Disorder, given the temporal relationship of the symptoms with the stressful history at work.

**Conclusions:**

Given their growing prevalence autoimmune causes, such as NMDA anti-receptor antibody encephalitis, should always be considered in the cases of neuropsychiatric alterations. It is very important to carry out a correct organic screening prior to the diagnosis of psychiatric pathology. It is also essential an adequate coordination between different medical departments for an accurate and comprehensive approach to the patient.

**Disclosure of Interest:**

None Declared

